# Efficacy of sulfur hexafluoride (SF_6_) versus perfluoropropane (C_3_F_8_) tamponade in large macular hole

**DOI:** 10.22336/rjo.2025.56

**Published:** 2025

**Authors:** Ashok Kumar, Sanjay Kumar Mishra, Vipin Rana, Vikas Ambiya, Pradeep Kumar, Poninder Kumar, Srujana Dubbaka, Vijay Kumar Sharma

**Affiliations:** 1 Department of Ophthalmology, Armed Forces Medical College, Pune, India; 2 Department of Ophthalmology, Army Hospital R&R, Delhi Cantt, India; 3 Department of Ophthalmology, Command Hospital, Kolkata, India

**Keywords:** Large macular hole, idiopathic, gas tamponade, SF_6_, C_3_F_8_, SF_6_ = sulfur hexafluoride, C_3_F_8_ = perfluoropropane, MLD = minimal linear diameter, μm = micron meter, IMH = Idiopathic macular hole, ILM = Internal limiting membrane, OCT = optical coherence tomography, FTMH = full-thickness macular hole, BCVA = best corrected visual acuity, logMAR = logarithm of the minimum angle of resolution, MHI = macular hole index

## Abstract

**Objective:**

To evaluate the efficacy of SF_6_ versus C_3_F_8_ gas tamponade for large idiopathic macular holes.

**Materials and methods:**

The study included 60 eyes with large macular holes managed with pars plana vitrectomy, inverted internal limiting membrane flap, and gas tamponade with either SF_6_ group (30 eyes of 30 patients) or C_3_F_8_ group (30 eyes of 30 patients). Based on minimal linear diameter (MLD), subjects were further classified into two groups: large (400 - <600 μm) or very large (>600 μm); compared for anatomical closure, improvement in best corrected visual acuity, and development of complications.

**Results:**

Both groups were comparable in baseline features of age, sex, etiology, and size of hole, with significantly larger MLD (708.2 ± 154.62 vs. 622.6 ± 114.08 μm; p=0.017) and a poorer macular hole index (0.36 ± 0.06 vs 0.31 ± 0.04; p=0.003) in the SF_6_ group. Macular hole closure was achieved in all eyes in the C_3_F_8_ group and 29 of 30 eyes in the SF_6_ group. Significant improvement was observed in vision in both groups from baseline at six months post-surgery. Cataract development/progression was observed in one patient in both groups, with no significant increase in intraocular pressure. In sub-groups of very large macular holes, despite significantly larger MLD and poorer macular hole index in the SF_6_ group, structural and functional outcomes were comparable to C_3_F_8_.

**Discussion:**

The management of large macular holes is a critical challenge as these conditions can severely impair central vision and quality of life for affected patients. Surgical intervention using tamponade agents is a standard approach to promote hole closure and retinal healing. Both SF_6_ and C_3_F_8_ have demonstrated effectiveness in promoting macular hole closure. While the evidence is somewhat mixed, several studies indicate that SF_6_ may be associated with quicker visual recovery in the early postoperative period. However, the long-term visual outcomes are often comparable between the two agents. In the present study, we compared the efficacy of both these tamponade agents for large and very large idiopathic macular holes.

**Conclusions:**

Both SF_6_ and C_3_F_8_ tamponades are equally effective in the treatment of large macular holes. However, despite poorer pre-operative prognostic factors like larger MLD and macular hole index, the efficacy and safety of SF_6_ gas tamponade are comparable to C_3_F_8_ tamponade.

## Introduction

A macular hole is a full-thickness defect of the neurosensory retina that results from the centrifugal movement of outer retinal layers under the effect of vitreo-macular forces at the foveal center [1]. Idiopathic macular hole (IMH) incidence ranges from 7.8 to 30 cases for every 100,000 citizens [2]. It commonly occurs in one eye only, but can be present bilaterally in up to 11.7% of cases. Since the introduction of pars plana vitrectomy as standard treatment for macular hole, various new techniques have been tried to improve visual and anatomical outcomes in macular hole patients [3]. Internal limiting membrane (ILM) peeling has become an essential component of macular hole surgery to achieve sustained improvement in visual acuity as well as an increased rate of anatomical success [4]. The inverted ILM flap technique as part of macular hole surgery showed superiority compared to the traditional ILM peeling technique in terms of both closure rate and visual outcomes, especially in large and recurrent macular holes [5].

The various tamponade materials are used to replace the vitreous to stabilize the retina and facilitate the flattening of the retinal profile, thereby increasing the success rate in closing the macular hole. Gas tamponade using perfluoropropane (C_3_F_8_) and sulfur hexafluoride (SF_6_) is the most frequently used and represents the first choice among various biomaterials. While the evidence is somewhat mixed, several studies suggest that SF_6_ may be associated with quicker visual recovery in the early postoperative period. However, the long-term visual outcomes are often comparable between the two agents. C_3_F_8_ offers a longer duration of tamponade compared to SF_6_, which may be advantageous in cases where prolonged support is needed. Although the procedure was initially described with the use of SF_6_, subsequent clinical trials used C_3_F_8_ with equally good results, further adding to the debate [6,7].

Kim et al. exhibited no significant difference regarding the anatomical hole closure rates between SF_6_ and C_3_F_8_ groups [8]. Similar results were also shown by Jackson et al. with various gas mixtures, including air, SF_6_, C_3_F_8_, and C_2_F_6_ as endo-tamponading agents, with no significant difference in macular hole closure rate [9]. Subgroup analysis was also done in this study to determine whether a longer-acting endo-tamponade agent enhances the anatomical outcome in holes with higher staging, larger diameters, or longer duration; interestingly, no difference was observed between the two groups, and both gases were equally effective irrespective of the stage, size, or duration of the hole. However, limitations of these studies include their retrospective design, lack of randomization, limited postsurgical follow-up, and a smaller sample of patients undergoing surgeries.

There is a definitive lack in the literature on comparative studies of the efficacy of SF_6_ versus C_3_F_8_, when combined with the inverted flap technique, especially for large-sized macular holes. So, we conducted this prospective, interventional study comparing SF_6_ to C_3_F_8_ as a tamponade agent combined with the inverted ILM flap technique in large macular holes and assessed in terms of anatomical, visual, and safety outcomes.

## Material and methods

This non-randomized prospective interventional study was conducted at three tertiary eye care referral centers spread across India over 2 years. Institutional ethical committee approval for the study was obtained at all centers, and the study was conducted in adherence to the tenets of the Declaration of Helsinki. Written informed consent was obtained from all participants before inclusion in the study.

**Fig. 1:**
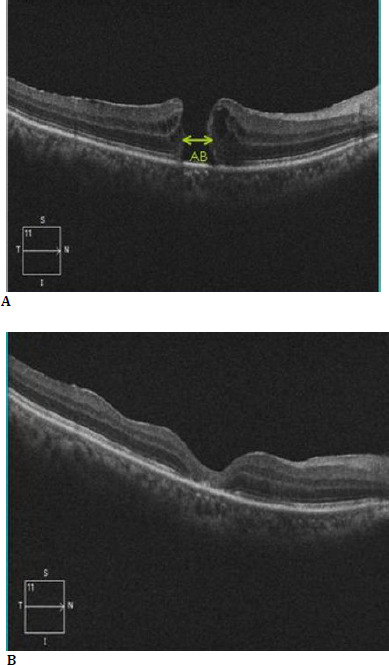
**A** Spectral domain Optical Coherence Tomogram (SD-OCT) of macula showing a full-thickness macular hole with AB line showing minimal macular hole width. **B**. SD-OCT of the macula of the same patient showing closure of the macular hole post-surgery, denoting successful anatomical closure

Inclusion criteria for patients to be eligible for participation in the study were: a pre-operative diagnosis of full-thickness macular hole (FTMH) confirmed by fundus examination and optical coherence tomography (OCT) images (**Fig. 1A**). Following International Vitreomacular Traction Study Group Classification, study subjects were further classified into two groups: large macular holes (>400 but <600 μm minimal linear diameter) and very large macular holes (>600 μm minimal linear diameter). The size of the macular hole was taken as the minimum hole width, drawing a line with the caliper parallel to the retinal pigment epithelium. The patients with secondary macular holes, associated retinal diseases like branch retinal vein occlusion, previous retinal detachment, recurrent macular holes, small hole (< 400 μm size), and inability to complete the mandatory 6-month post-operative follow-up or maintain post-operative prone positioning were excluded from the present study. A total of 60 patients were included in the study over two years, with 20 patients at each of the three participating centers.

Before surgery as well as at 1 week, 1 month, 3 and 6 months after surgery, all patients underwent a complete ophthalmic examination, including best corrected visual acuity (BCVA), Goldman applanation tonometry, fundus examination using spectral domain OCT (Carl Zeiss Meditec, Inc., 5160 Hacienda Drive, Dublin, CA 94568 USA) using macular cube (6.0 × 6.0 mm; 512 × 128) and high-definition scans. All macular hole surgeries were conducted under peribulbar block by the same experienced retinal surgeon at each centre using a uniform surgical technique. All patients were operated on using standard 25-gauge pars plana vitrectomy using the Constellation vitrectomy system (Alcon Labs Inc., Fort Worth, TX). Triamcinolone-assisted posterior vitreous separation was done to ensure complete vitreous removal. Brilliant blue G (0.5%) dye-assisted ILM peeling was initiated using intravitreal ILM forceps (Grieshaber, Alcon, USA) with at least 2 Disc diameter-sized ILM peels in all cases, with the fovea as the centre, extending up to disc margins, with the inverted flap technique in all cases, followed by fluid-air exchange. The gas tamponade was performed using either Perfluoropropane (C_3_F_8_) as 14% or 20% sulfur hexafluoride (SF_6_) gas with prone positioning for at least 72 hours after surgery. Sclerotomy sites were self-sealing, but if any leakage was noted, then they were sutured. Topical antibiotics, cycloplegics, and steroids were prescribed to all patients postoperatively and gradually tapered over the next four weeks.

The successful anatomical closure of the macular hole confirmed on SD-OCT post-surgical procedure was considered as the principal outcome measure of efficacy of gas tamponade images (**Fig. 1B**). The improvement in BCVA following macular hole closure was taken as a secondary outcome measure of effectiveness. Development and/or progression of cataract was defined by new onset of cataract or increase in nuclear sclerosis by >1 grade or drop in visual acuity attributable to cataract by > 2 Snellen’s lines. The study subjects were also monitored at each follow-up visit for an increase in intraocular pressure or recurrence of macular hole over the next six months.

### Statistical analysis

The Snellen BCVA was converted to the logarithm of the minimum angle of resolution (logMAR) equivalent for statistical analysis. An independent sample t-test was used to compare the means of parameters between the groups. The changes from baseline in BCVA were analyzed using the Wilcoxon signed-rank test. A p-value of <0.05 was considered statistically significant.

## Results

A total of 60 patients were recruited in this study, with 30 each randomized into the SF_6_ and C_3_F_8_ groups. The two groups were comparable in baseline characteristics; in terms of age, sex, BCVA, etiology of macular hole, lens status of patients, and distribution of size of macular hole (large vs. very large). However, there was a statistically significant difference in minimal linear diameter (MLD) and macular hole index (MHI) between the SF_6_ group compared with the C_3_F_8_ group (p=0.017; p=0.003) (**Table 1**).

**Table 1:** Baseline demographic characteristics and clinical parameters of both study arms

Baseline Characteristics	SF_6_ group (n=30)	C_3_F_8_ (n=30)	p-value
Age (years + SD)	58.1 ± 14.91	61.5 ± 16.98	0.075
Sex (M:F)	10:20	15:15	0.33
BCVA (logMAR)	1.48 ± 0.22	1.43 ± 0.16	0.34
Minimal Linear Diameter (μm)	708.2 ± 154.62	622.6 ± 114.08	**0.017**
Large/Very Large (numbers)	6:24	9:21	0.45
Macular Hole index (MHI)	0.36 ± 0.06	0.31 ± 0.04	**0.003**
Lens Status (Phakic/Pseudo phakic)	22:8	25:5	0.67

### Anatomical outcome

The outcome of surgical intervention in the form of pars plana vitrectomy with ILM peeling (inverted flap technique) and gas tamponade at the last follow-up is shown in **Table 2**. The successful macular hole closure was achieved in all patients in the C_3_F_8_ group and 29 of 30 patients in the SF_6_ group with no statistical difference in success rate at last follow-up (p=0.89). Moreover, a favorable outcome in the form of type 1 closure of macular hole was achieved in both study arms; although a greater number of patients in the C_3_F_8_ group achieved type 1 closure (p=0.12).

**Table 2:** Post-surgical intervention outcome in both study arms at the last follow-up

Characteristics	SF_6_ group (n=30)	C_3_F_8_ (n=30)	p-value
Macular Hole closed/Not closed	29:01	30:0	0.89
Type of closure achieved (Type 1/Type 2)	24:6	28:2	0.12

### Change in BCVA

There was a statistically significant improvement in BCVA in both groups from baseline at six months post-surgical intervention: in group SF_6_, 1.48 ± 0.22 to 0.59 ± 0.13 logMAR (p<0.01) and in group C_3_F_8_, 1.43 ± 0.16 to 0.61 ± 0.08 logMAR (p<0.01). However, there was no statistically significant difference in the improvement of BCVA between the two study groups at one, three, and six-month follow-up (p=0.64).

### Adverse effects/complications of the surgical procedure

**Fig. 2:**
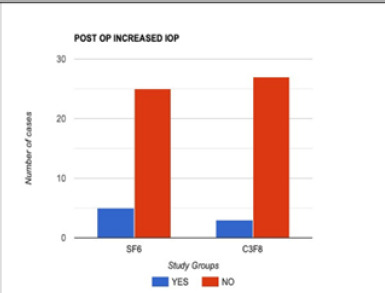
Comparison of the increase in IOP in the two groups

**Fig. 3:**
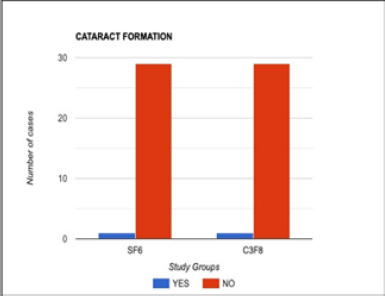
Comparison of postoperative cataract formation in two groups

There was no statistically significant complication of the surgical procedure in the two subgroups, which shows that surgery had comparable outcomes in the closure of macular holes in both study arms. However, the number of patients experiencing a postoperative rise in intraocular pressure was slightly greater in the C_3_F_8_ group (**Fig. 2**), which was adequately controlled with short-term anti-glaucoma medication and a comparable number of cataract formations in both sub-groups (**Fig. 3**) (**Table 3**).

**Table 3:** Post-surgical intervention complications in both study arms at the last follow-up

Characteristics	SF_6_ (n=6)	C_3_F_8_ (n=9)	p-value
Age	**63.5 ± 17.75**	**66.22 ± 9.48**	0.70
BCVA (Pre-op)	**1.62 ± 0.15**	**1.54 ± 0.14**	0.07
Minimal Linear Diameter	**534.5 ± 51.13**	**486.67 ± 57.65**	0.12
Post-Surgical BCVA at 1 month	**0.8 ± 0.22**	**0.73 ± 0.14**	0.48
Post-Surgical BCVA at 3 months	**0.5 ± 0.17**	**0.61 ± 0.11**	0.06
Post-Surgical BCVA at 6 months	**0.5 ± 0.17**	**0.62 ± 0.07**	0.06
MHI	**0.37 ± 0.08**	**0.30 ± 0.04**	**0.04**

### Sub-group analysis of large and very large macular holes

Following the International Vitreomacular Traction Study Group Classification, study subjects were further classified into two groups: large macular holes (>400 but <600 μm minimal linear diameter) and very large macular holes (>600 μm minimal linear diameter). The size of the macular hole was taken as the minimum hole width, drawing a line with the caliper parallel to the retinal pigment epithelium.

The two groups were comparable in baseline characteristics; in terms of age, BCVA (preoperative), and postoperative BCVA at 1, 3, and 6 months. However, there was a statistically significant difference in the macular hole index (MHI) between the SF_6_ group compared with the C_3_F_8_ group in both the large and very large holes group (p=0.04, p=0.003) (**Table 4, 5**). Being a short-term tamponade as compared to C_3_F_8_ gas, SF_6_ tamponade had a comparable surgical outcome despite having a larger minimal linear diameter as well as a poorer macular hole index.

**Table 4:** Baseline demographic characteristics and postoperative outcome analysis of the large holes study arm

Characteristics	SF_6_ group (n=30)	C_3_F_8_ (n=30)	p-value
Recurrence of macular hole (Yes/No;1/2)	-	-	-
MHI	0.36 ± 0.06	0.31 ± 0.04	**0.003**
Phakic/Pseudophakic (1/2)	22:8	25:5	0.67
Increased IOP Yes/No (1/2)	5:25	3:27	0.51
Development/Progression of Cataract Yes/No (1/2)	1:29	1:29	0.45
**Characteristics**	**SF_6_ group (n=30)**	**C_3_F_8_ (n=30)**	**p-value**
Recurrence of macular hole (Yes/No;1/2)	-	-	-
MHI	0.36 ± 0.06	0.31 ± 0.04	**0.003**
Phakic/Pseudophakic (1/2)	22:8	25:5	0.67
Increased IOP Yes/No (1/2)	5:25	3:27	0.51
Development/Progression of Cataract Yes/No (1/2)	1:29	1:29	0.45

**Table 5:** Baseline demographic characteristics and post-operative outcome analysis of the very large holes study arm

Characteristics	SF_6_ (n=24)	C_3_F_8_ (n=21)	p-value
Age	**58.75 ± 14.23**	63.76 **±** 5.72	0.06
BCVA (Pre-op)	**1.45 ± 0.22**	**1.47 ± 0.15**	0.65
Minimal Linear Diameter	**751.63 ± 140.49**	**680.86 ± 75.25**	**0.04**
Post-Surgical BCVA at 1 month	**0.70 ± 0.17**	**0.8 ± 0.24**	0.13
Post-Surgical BCVA at 3 months	**0.7 ± 0.16**	**0.62 ± 0.09**	0.06
Post-Surgical BCVA at 6 months	**0.62 ± 0.12**	**0.6 ± 0.09**	0.60
MHI	**0.36 ± 0.05**	**0.31 ± 0.05**	**0.003**

## Discussion

Macular hole (MH) is a full-thickness defect of the neurosensory retina that results from centrifugal movement of outer retinal layers under the effect of vitreo-macular forces and commonly occurs unilaterally. However, it can occur bilaterally in up to 11.7% of cases. Macular hole can be further divided into small, medium, and large depending on the minimal linear diameter of the hole, with treatment remaining essentially surgical in all subtypes [3].

The management of large macular holes is a critical challenge as these conditions can severely impair central vision and quality of life for affected patients. Surgical intervention using tamponade agents is a standard approach to promote hole closure and retinal healing. Both SF_6_ and C_3_F_8_ have demonstrated effectiveness in promoting macular hole closure. The literature suggests that both agents have high closure rates [10-18]. While the evidence is somewhat mixed, several studies indicate that SF_6_ may be associated with quicker visual recovery in the early postoperative period [8,15]. However, the long-term visual outcomes are often comparable between the two agents. C_3_F_8_ offers a longer duration of tamponade compared to SF_6_, which may be advantageous in cases where prolonged support is needed, such as in complex retinal detachment. Both SF_6_ and C_3_F_8_ are generally well-tolerated, but some studies indicate potential complications associated with these agents [19-21]. Most of these studies were performed on idiopathic macular holes, and they have not considered large macular holes.

In a retrospective comparative study, Bori et al. compared the effectiveness of pars plana vitrectomy (PPV) with either silicone oil or gas tamponade for the treatment of traumatic macular holes and concluded that C_3_F_8_ gas provided more effective tamponade than silicone oil in achieving initial closure of traumatic macular holes [19]. They also concluded that cases of TMHs should be observed for spontaneous closure. PPV with ILM peeling should be conducted for non-closing cases. However, silicon oil can more commonly lead to cataract formation, elevated intraocular pressure, and the risk of reopening of macular holes in some cases.

In a randomized prospective study on early visual and anatomical outcomes with sulfur hexafluoride (SF_6_) or perfluoro propane (C_3_F_8_) tamponade for macular hole repair, Casini G et al. concluded that visual outcomes were similar in eyes treated with either SF_6_ or C_3_F_8_, independently of the stage of the macular hole and age, gender not being an influencing factor for the prognosis [20].

In our study, the demographic profile, baseline characteristics, and postoperative outcome were comparable with those of this study by Casini et al. Also, there were no statistically significant postoperative complications like cataract formation and intraocular pressure rise. The results of our study were also comparable to those of Modi et al., who also observed that the macular hole closure rate was similar with sulfur hexafluoride and perfluoropropane, irrespective of hole size, stage, or duration. However, sulfur hexafluoride exhibited a decreased incidence of cataract and ocular hypertension with shorter tamponade duration [21].

In our study, we also compared the large and very large macular hole groups in terms of their baseline characteristics, gas tamponade, and postoperative outcomes. The two groups were comparable in baseline characteristics; in terms of age, BCVA (preoperative), and postoperative BCVA at 1, 3, and 6 months. However, there was a statistically significant difference in macular hole index (MHI) as well as minimum linear diameter between the SF_6_ group compared with the C_3_F_8_ group, despite being equally efficacious in post-surgical anatomical closure and functional improvement.

The strength of this study is in analyzing and comparing the safety and efficacy of the two commonly used gas tamponades, C_3_F_8_ and SF_6_, in idiopathic large macular holes with a probable poor anatomical closure rate due to large size. This study had certain limitations, such as a relatively small no of subjects included in both groups. However, this study might have been limited to patients with large macular holes (> 400 μm). Still, it had the advantage of recruiting patients from all ethnic and demographic populations of the sub-continent, as it was conducted in multiple centers. To the best of our knowledge, perhaps this is the first study exhibiting a comparable efficacy of SF_6_ gas tamponade in comparison to C_3_F_8_ gas tamponade despite having poorer preoperative prognostic factors in the form of minimum linear diameter and macular hole index.

## Conclusion

To conclude, both sulfur hexafluoride (SF_6_) and perfluoropropane (C_3_F_8_) gas tamponade are equally effective in the treatment of large macular holes. However, despite having poorer preoperative prognostic factors in the form of larger minimal linear diameter and macular hole index, the efficacy and safety of SF_6_ gas tamponade were comparable to C_3_F_8_ tamponade. Therefore, SF_6_ gas tamponade may be a preferable tamponade agent to C_3_F_8_ even in large and very large macular holes.
